# Biophysics Austria: 65 years history

**DOI:** 10.1007/s00249-026-01866-z

**Published:** 2026-09-22

**Authors:** Elena E. Pohl, Klaus Groschner

**Affiliations:** 1 Medical Physics and Biophysics, Department of Biological Sciences and Pathobiology, University of Veterinary Medicine, Vienna, 1210 Austria; 2 Division of Medical Physics and Biophysics, Medical University of Graz, Graz, 8010 Austria

**Keywords:** European biophysical societies association, International union for pure and applied biophysics, Austrian biophysical society, European biophysics congress

## Abstract

The Austrian Biophysical Society (Biophysics Austria) was founded in 1961, making it one of the oldest national biophysical societies in Europe. It hosted the first European Meeting in Biophysics in Baden near Vienna in 1971 and celebrated its 60th anniversary by hosting the 13th European Biophysics Congress in Vienna as a hybrid event during the COVID-19 pandemic in 2021. In 2026, the society is celebrating its 65th anniversary. This review reflects on the society´s history and highlights events, milestones, and people who have contributed to its development.

## Introduction

Fifty-five years ago, in September 1971, the first European Biophysics Societies’ Association (EBSA) conference took place in Austria, in Baden near Vienna. Although the EBSA itself was not formally established until 1984 (Seddon and Watts 2025), this meeting is considered as the first in the series of EBSA congresses. The meeting was organized by the Austrian Biophysical Society, which was only ten years old at the time. Founded in 1961 as the “Austrian Society for Pure and Applied Biophysics,” it is one of the oldest Austrian academic societies in the life sciences.

The trigger to establish a national scientific association was the first meeting of the International Union for Pure and Applied Biophysics in Stockholm, which was also founded in 1961. From the beginning, the Austrian Society has been committed to interdisciplinary research, interconnecting biology, medicine, physics and chemistry. Over the years, the society has experienced ups and downs but has grown into a stable platform that supports biophysical research and fosters communication between biophysicists in Austria and beyond.

In this historical review dedicated to the 65th anniversary of the Austrian Biophysical Society we take the opportunity to reflect on the society´s history and highlight some of the people who have contributed to its development.

### The early years of the society

**Fig. 1:**
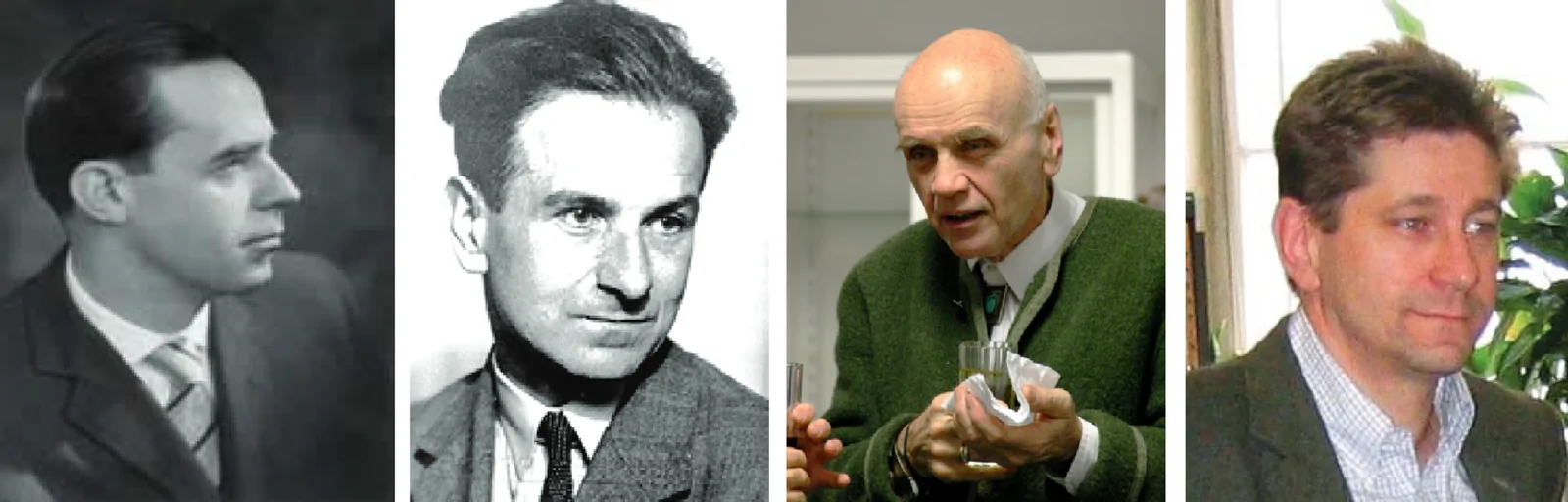
(**A**) Hans Bornschein (1920–1979). © Archiv of the University of Vienna . (**B**) Engelbert Broda (1910–1983). © Austrian Central Library for Physics. (**C**) Helmut Horvarth, president of the Society 1998–2004. © Horvath Private Photo Album. (**D**) Martin Hohenegger, president of the Society 2004–2010. © Martin Hohenegger, Private Photo Album.

In 1961, Drs. Karl Kaindl and Helmut Knötig, sparked the formation of an initiative committee after returning from the first meeting of the International Union for Pure and Applied Biophysics (IUPAB) in Stockholm. They quickly organized a general assembly on December 20, 1961, to establish the Austrian Biophysical Society. The assembly elaborated the first constitution and elected Prof. Dr. Hans Bornschein (Fig. 1), the founder of the Institute of General and Comparative Physiology at the University of Vienna, as the first President. Bornschein was internationally recognized for his pioneering research in the fields of biological cybernetics and sensory physiology, particularly for his analysis of signal transduction mechanisms in the retina. The society was an Austrian equivalent of IUPAB and was called the “Österreichische Gesellschaft für reine und angewandte Biophysik“ (Austrian Society for Pure and Applied Biophysics). The society was quite active from its early days, organizing and participating in joint meetings with the other biophysical societies. Only three years after its foundation, in September 1964, Vienna hosted a joint meeting involving the German Society for Biophysics the Swiss Society for Radiation Biology and the Austrian Society for Pure and Applied Biophysics (von Bertalanffy 1965). These activities culminated in the organization of the second IUPAB meeting (5–9 September 1966) in Vienna and the first European Biophysical Societies’ Association (EBSA) congress (14–17 September 1971) in Baden near Vienna. At that time, the society changed its name to Österreichische Biophysikalische Gesellschaft (ÖBG, Austrian Biophysical Society). National and international speakers were regularly invited to cover the broad field of biophysics and to foster our ties to the community of biophysicists worldwide.

The biological and biophysical consequences of nuclear radiation were a major societal issue in Austria in the 1970s, culminating in the 1978 referendum against nuclear power plants. Under the leadership of the third ÖBG president, Engelbert Broda (Fig. 1; Table 1), the ÖBG was heavily involved in grounding this discussion in science. Broda´s scientific work was strongly connected to bioenergetics, which is one of the central themes of early biophysics. From 1938 to 1947 Broda worked on rhodopsin at University College London, in the United Kingdom, as well as on radioactivity and nuclear fusion at the Cavendish Laboratory in Cambridge (https://zbphysik.univie.ac.at/broda/). Examining the research fields of the societies’ presidents (Table 1) reveals the diversity of research areas addressed by our society.

**Table 1:** Presidents of Biophysics Austria 1961–2026

Acting period	Presidents	Institution	Discipline, research focus and activities
1961–1967	Hans **Bornschein**	Institute of Physiology, University of Vienna	**Physiology**: Signal transduction in retina, biological cybernetics and Bioinformatics
1967–1970	Johann **Schedling**	Institute of Medical Physics, University of Vienna	**Environmental Physics**: air pollution, radioactivity and environment
1970–1972	Engelbert **Broda**	Institute of Physical Chemistry, University of Vienna	**Nuclear Physics and Bioenergetics**; Environmental- and Peace activist
1972–1981	Richard **Brunner**	Institute of Chemistry, Vienna University of Technology, Vienna	**Biotechnology and Microbiology**: Fermentation, Brewing Science, Antibiotics
1981–1987	Maximilian **Hohenegger**	Institute of Experimental Pathology, University of Vienna	**Experimental Pathology**: Ion homeostasis, Metabolism
1987–1998	Wolfgang **Seiler**	Institute of Medical Physics, University of Vienna	**Physics**: Radioactivity
1998–2004	Helmuth **Horvath**	Institute of Experimental Physics, University of Vienna	**Environmental Physics**: Aerosoles
2004–2010	Martin **Hohenegger**	Institute of Pharmacology, Medical University, Vienna	**Pharmacology**: Muscle physiology, ryanodine receptors
2010–2013	Georg **Pabst**	Institute of Biophysics, Austrian Academy of Sciences, Graz	**Membrane Biophysics**: lipid organization in membranes, protein-lipid interactions
2013–2015	Gerhard **Schütz**	Institute of Applied Physics and Biophysics, Vienna University of Technology, Vienna	**Membrane Biophysics**: single molecule spectroscopy and imaging, membrane nanostructures
2015–2017	Klaus **Groschner**	Division of Medical Physics and Biophysics, Medical University of Graz	**Ion channel Biophysics**: TRP channels, photopharmacology
2017–2019	Thomas **Stockner**	Institute of Pharmacology, Medical University of Vienna	**MD-simulations**: structure-function relations in ion channels and transporters
2019–2022	Birgit **Plochberger**	FH Oberösterreich, Linz	**Physical Biochemistry**: single molecular fluorescence and atomic force microscopy, lipoproteins
2022–2024	Elena E. **Pohl**	Medical Physics and Biophysics, University of Veterinary Medicine Vienna	**Membrane Biophysics**: mitochondrial membrane proteins, cancer, immunometabolism
2024–2026	Bernhard **Flucher**	Physiology and Medical Physics, Medical University of Innsbruck	**Biophysics**: Calcium channels, neuro-muscular physiology
2026–2028	Peter **Pohl**	Institute of Biophysics, Johannes Kepler University, Linz	**Biophysics of membrane transport**: proton and water transport, protein translocation, membrane mechanics

### First EBSA congress

**Fig. 2:**
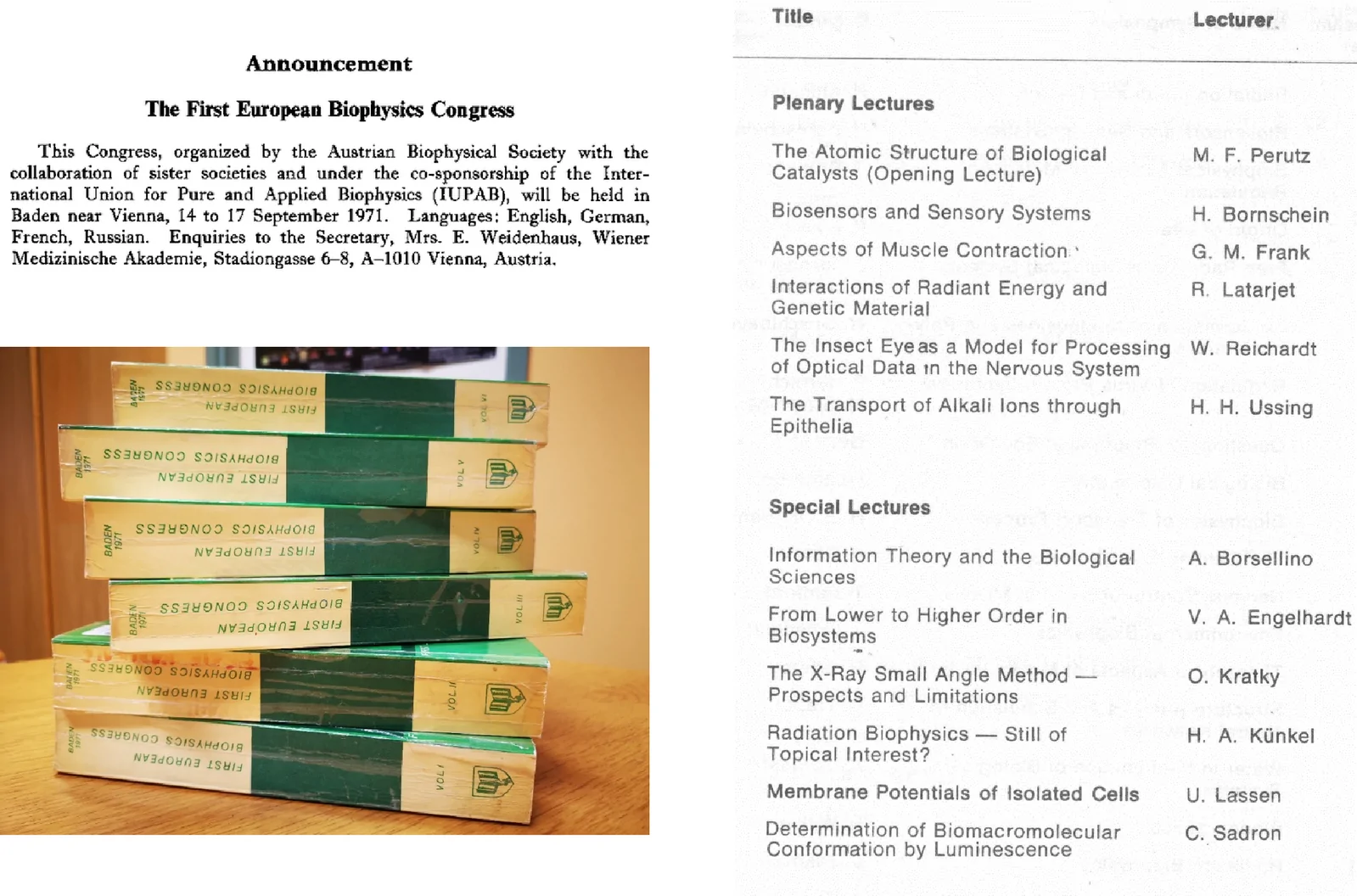
The first European Biophysical Congress in Baden near Vienna, 14–17 September 1971. (**A**) Announcement in Quarterly Reviews of Biophysics (1970), 3, 3 377–381 (**B**) Program and Abstracts. (**C**) Plenary lectures.

Undoubtedly, the most significant event in the early history of the society was the first European Biophysics Meeting, which was hosted by Biophysics Austria in Baden, near Vienna, in 1971 (Fig. 2). This event was organized in collaboration with sister societies, was co-sponsored by the IUPAB and was a first successful attempt to bring national biophysical communities together. The conference featured 19 symposia that covered a broad spectrum of biophysical topics, ranging from proteins and membranes to theoretical and environmental biophysics. The opening lecture was given by Austrian chemist Max F. Perutz, who won the Nobel Prize in 1962 for his work on the structures of hemoglobin and myoglobin. The multi-volume proceedings were edited by Broda, Locker and Springer-Lederer, and published by Verlag der Wiener Medizinischen Akademie on behalf of the Austrian Biophysical Society (Broda et al. 1971). The six thematic volumes were entitled (1) Proteins and Nucleotides: Structure, Function, and Biosynthesis; (2) Radiation Biophysics and Free Radicals; (3) Membranes and Transport; (4) Photosynthesis, Bioenergetics, Regulation, and the Origin of Life; (5) Cells and Organs: Nervous, Sensory, and Contractile Systems; and (6) Theoretical Molecular Biology, Biomechanics, Biomathematics, Environmental Biophysics, Techniques, and Education (Fig. 2). These volumes demonstrated the full scope of the biophysical issues discussed at the first congress. Each of these volumes corresponded to a major topical section of the congress and contained the names of speakers, titles of talks, and the structure of sessions for that topic.

### The time between the first and the 13th EBSA conference

Over the years, the Austrian Biophysical Society attracted members from a variety of scientific backgrounds, including physics, biology, and medicine. The society´s research interests have ranged from bioinformatics, environmental physics, biotechnology, bioenergetics, and pharmacology to, more recently, molecular biophysics and computational biology. This diversity reflects an understanding of biophysics as a genuinely multidisciplinary field. From 1972 to 1981, the society was led by Richard Brunner, who is renowned for developing the first oral penicillin (Brunner 1963), as well as a biotech process for producing alcohol-free beer.

During the 1980s and 1990s, Austria developed several important centers of biophysical research, particularly in Vienna, Linz, Graz and Innsbruck. Excellent biophysicists established their laboratories during this period. Notable figures include Hansgeorg Schindler and Peter Laggner. Schindler founded the first Institute of Biophysics in Austria, located in Linz. The institute is currently led by Peter Pohl and Peter Hinterdorfer. Schindler also initiated single-molecule research in Austria (Hinterdorfer et al. 1996; Schmidt et al. 1996), which led to the establishment of the Annual Linz Winter Workshop in 1999. This conference is now headed by Peter Hinterdorfer, Gerhard Schütz and Yoo Jin Oh and has developed into a renowned international meeting that annually brings together 150–200 scientists from diverse disciplines to explore the frontiers of single-molecule research, nanoscience, and nanomedicine on a unique interdisciplinary platform for researchers from academia and industry. Schindler (1943–2001) was honored through the society’s Hansgeorg Schindler Young Investigator Award. The first winner of this award, sponsored by Tecan, was Klaus Schicker, a postdoctoral fellow at the Medical University of Vienna, for his work on the characterization of the human serotonin transporter (hSERT) protein in 2012 (Biophysics News, No 5, 2012). Another key figure was Peter Laggner, a world leading pioneer in small-angle X-ray scattering on low density lipoproteins (LDL) and biomembranes (Laggner 1994) and director of the Austrian Academy of Sciences Institute of Biophysics and Nanosystems Research in Graz. The latter was founded by Otto Kratky as Institute for X-Ray Fine Structure Research of the Austrian Academy of Sciences. Our society’s outreach was enhanced by appealing to researchers internationally, including Austrians who pursued their careers abroad, and colleagues who obtained their PhD or postdoctoral training in Austria.

From 1980 to 2000, Austria’s scientific landscape diversified, with many small scientific societies emerging. This may explain why an interdisciplinary field such as biophysics received less attention. Helmuth Horvath, president of the Society from 1998 to 2004 (Fig. 1), adapted the constitution to reflect contemporary requirements, introducing new ideas and members to the society. His successor, Martin Hohenegger (Fig. 1), successfully established the Austrian membership in EBSA in 2009 and organized the first joint meeting with the Austrian Society for Molecular Biology and Biotechnology (ÖGMBT) the following year.

**Fig. 3:**
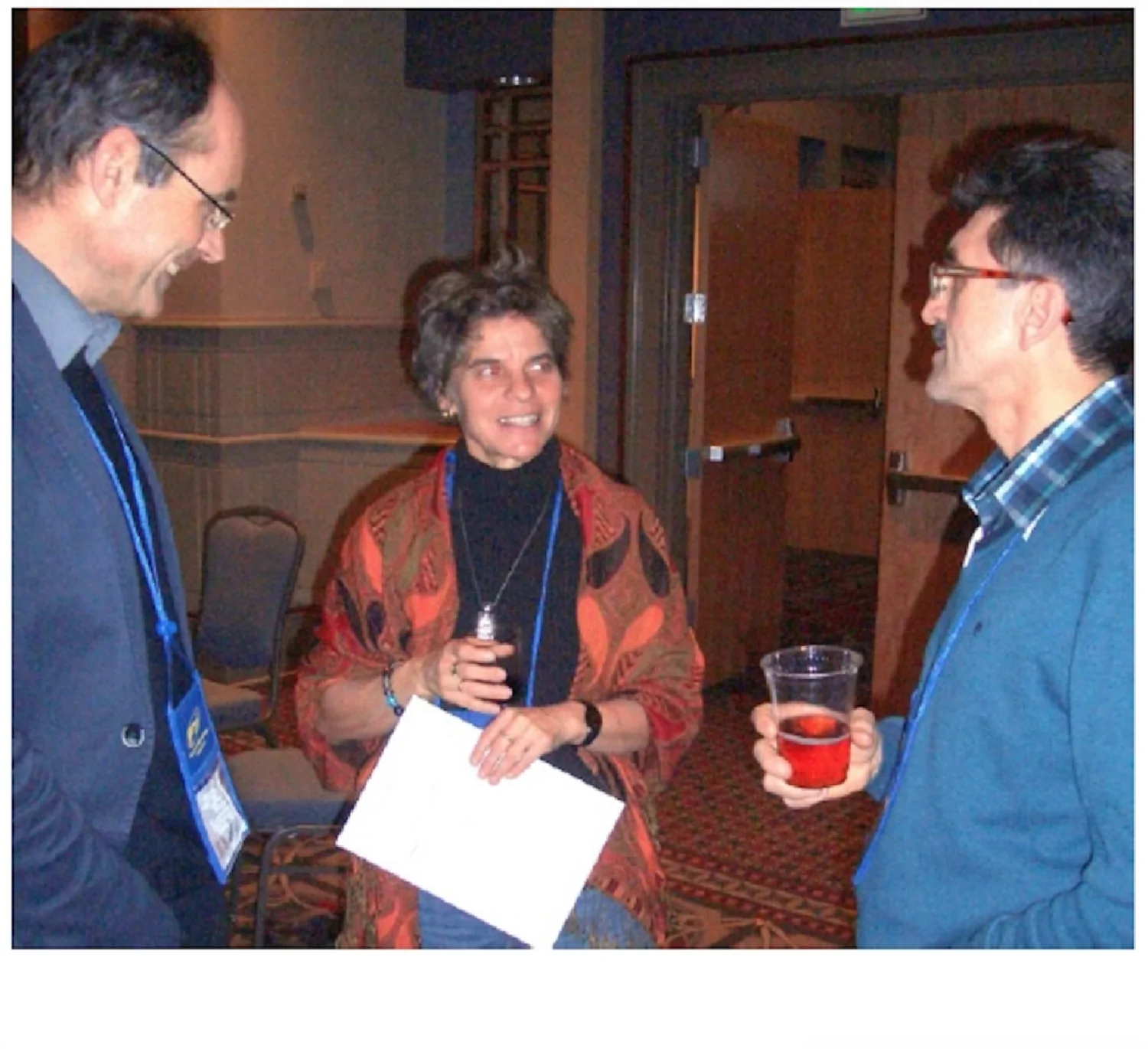
Karolin Luger - the Biophysical Society National Lecturer in Philadelphia, USA, 2013 - at the Austrian Biophysical Society Mixer. Left, J Striessnig, right, K. Groschner. ©Klaus Groschner; private photo album.

Thus, Biophysics Austria has developed into an open society that understands itself as part of the global biophysical community. This development was essentially promoted during the presidencies of Georg Pabst and Gerhard Schütz through continuous improvement of (digital) communication with society members, regular updates on society issues via a newsletter, and the establishment of the most attractive get-together of the Biophysics Austria family: the Biophysics Austria Mixer at the Annual Meetings of the Biophysical Society of America. This regular get-together of the Society in the US fostered the interactions between Austrian biophysicists at an international level (Fig. 3).

The successfully established concept of Biophysics Austria as an efficient platform for national communication combined with activities to increase international visibility of the Austrian biophysical community, was further developed during the presidency of Klaus Groschner and Thomas Stockner (2015–2019). In this period, the society embraced and supported the rapidly evolving and expanding field of computational biology, which opened entirely new aspects of biophysical research.

Recently, Biophysics Austria was led by its first female presidents, Birgit Plochberger (2019–2022) and Elena E. Pohl (2022–2024). Their leadership coincided with two particularly challenging and transformative periods for society: the COVID-19 pandemic and the organization of the EBSA Congress 2021 in Vienna. Their presidencies marked an important step towards greater gender balance and demonstrated the society’s ability to successfully navigate major challenges while strengthening its international visibility. The Society also strengthened its support of the next generation of scientists by launching a new initiative that awards travel scholarships to young members to attend EBSA congresses, starting with the EBSA Congress in Stockholm in 2023.

### EBSA 2021 in Vienna

In 2017, Biophysics Austria won the bid to host the biennial meeting of the European Biophysical Societies’ Association in Vienna in 2021.

**Fig. 4:**
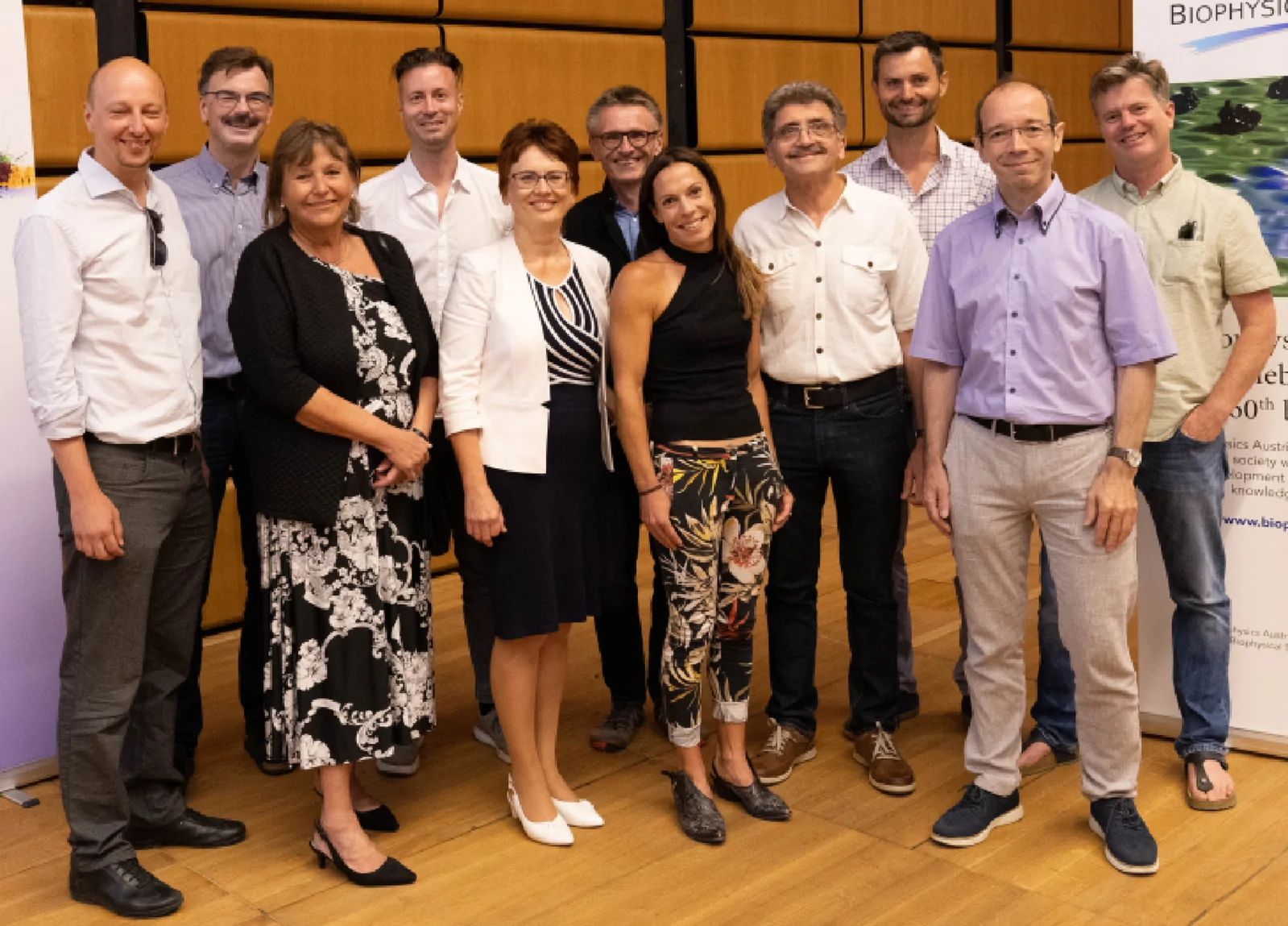
Local organization committee of the 13th European Biophysical Congress in Vienna gives an excellent snapshot of the modern Austrian biophysical community. From left to right: Rainer Schindl, Peter Pohl, Ruth Prassl, Erik Reimhult, Elena E. Pohl, Gerhard Schütz, Birgit Plochberger, Klaus Groschner, Andreas Horner, Thomas Stockner, Georg Pabst. © EBSA 2021Congress Archiv.

EBSA 2021 was the first in-person congress to take place at the Austria Center Vienna since the outbreak of the global SARS-CoV-2 pandemic and was one of the few large in-person meetings worldwide. Congress chair, Elena E. Pohl, was supported by the local organizing committee (Fig. 4) and the EBSA executive committee.

The 2021 meeting was unique in many ways. For the first time in the history of biophysical congresses, the 13th EBSA meeting took place in hybrid form, both on-site and online. This allowed interested biophysicists worldwide, regardless of the existing travel restrictions, to participate in the event. Four hundred thirty-five participants from 37 countries attended in person. Around 170 speakers shared their latest research findings.

**Fig. 5:**
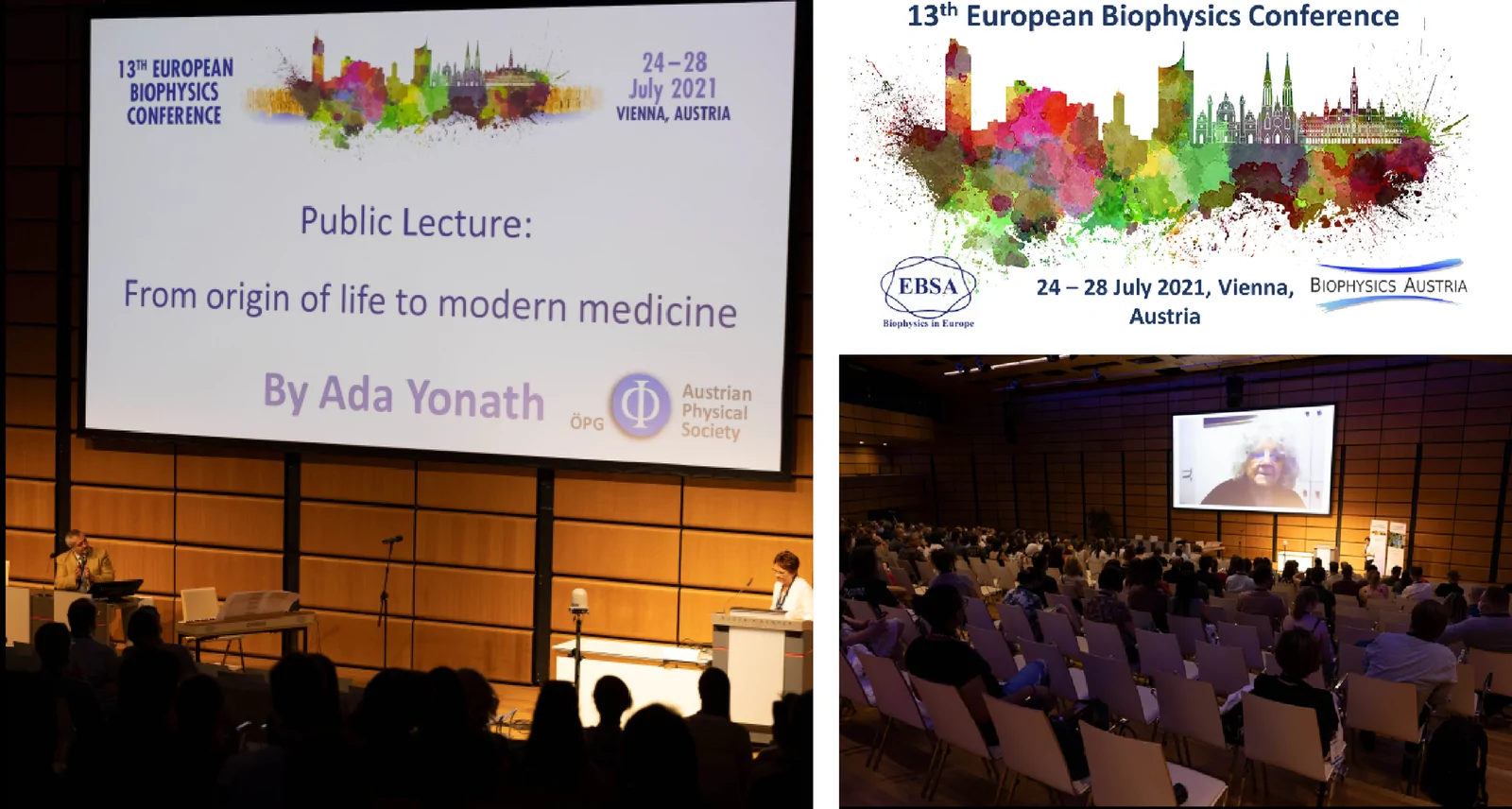
The 13th European Biophysical Congress in Vienna. The opening lecture was given by online Ada E. Yonath. © EBSA 2021 Congress Archiv. The logo of the congress is shown above, on the right.

For the first time, the congress began with a public lecture open to anyone interested in science. Nobel laureate Ada Yonath honored Erwin Schrödinger’s contributions to the life sciences by sharing her perspective on the enduring question, What Is Life? (Fig. 5). Her lecture was particularly fitting, as Schrödinger’s seminal book of the same title was also written for a broad audience. The event fell on the 60th anniversary of the death of the Austrian physicist and Nobel laureate.

**Fig. 6:**
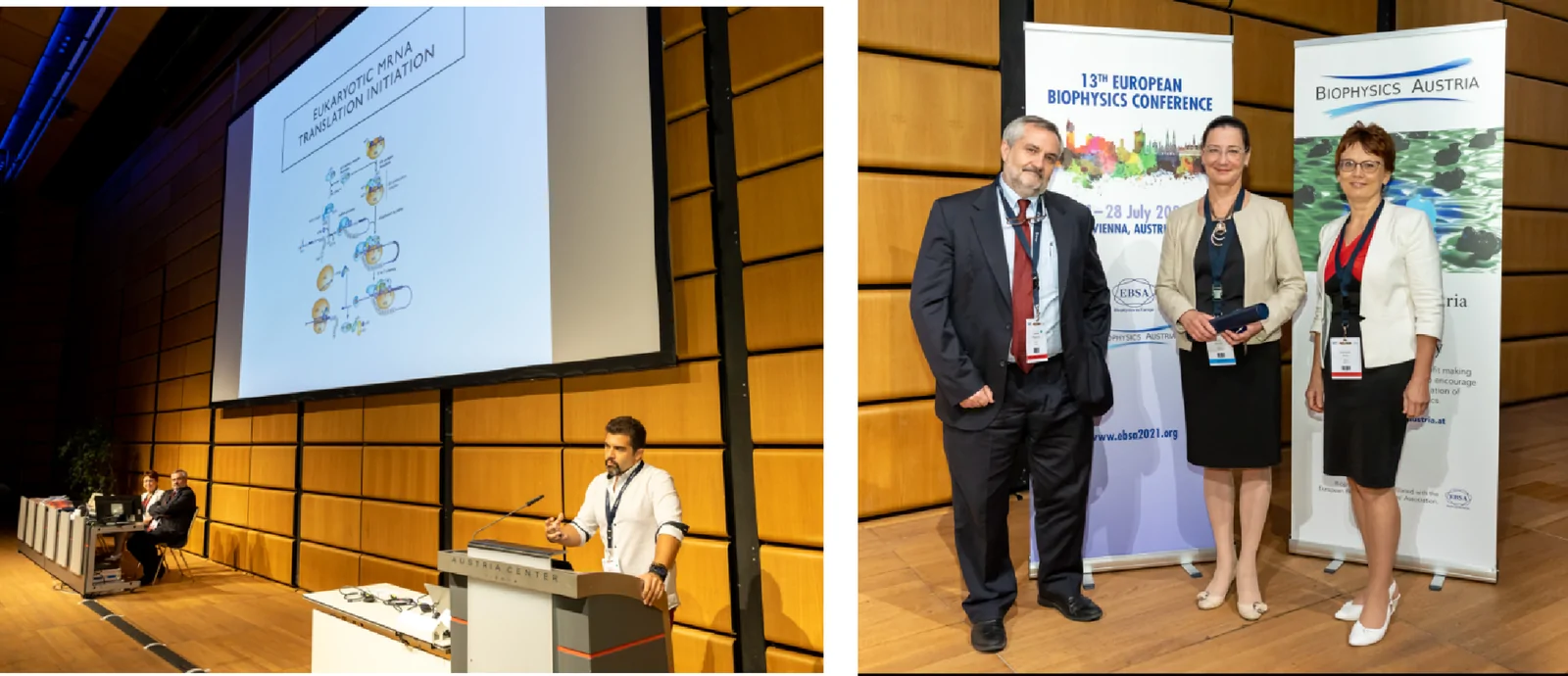
The 13th European Biophysical Congress in Vienna. **(A)** The presentation of EBSA Young Investigator Awardee Yaser Hashem, University of Bordeaux **(B)** Elina Ikonen (University of Helsinki, center) receives Avanti Lipids Award. Jesus Perez-Gil (EBSA president 2019-2021, left), Elena E. Pohl (congress chair, right). Photograph by Thomas Lerch, © EBSA 2021 Congress Archiv.

World´s leading biophysicists - Thomas Südhof (Nobel Prize for physiology and medicine, 2013), Francisco Bezanilla, Raimund Dutzler, Gerhard Hummer, Karolin Luger, Marina Rodnina, Carol Robinson gave plenary lectures about the state of the art in different biophysics research areas. Yaser Hashem received the EBSA Young Investigator Award for his contributions to cryo-electron microscopy and structural biology, while Elina Ikonen received the Avanti Polar Lipids/EBSA Award for her seminal work on the structure and function of biological membranes (Fig. 6). Most participants were excited to attend a scientific conference again after a long time. The relaxed and inspiring environment, spontaneous interactions, new acquaintances, and the ability to observe body language during group discussions all facilitated more efficient exchanges. The face-to-face setting made it far easier to establish new and unexpected collaborations, and to network with people from different fields whom you might not otherwise meet.

For the first time, new digital technologies enabled an additional 267 scientists, who could not come to Vienna to attend in person, to participate actively. As a result, we had lecturers who were able to present their latest findings even though they had to stay in their home country. The evening poster discussions over snacks and wine were a great success.

The success of the meeting was reflected in the participants’ feedback. As one professor from the United Kingdom put it: “This was by far and away the most difficult congress to organize of all time, but it was a huge success. Anyone who wasn’t there missed a lot!”

### Biennial meetings of Austrian Biophysical Society

**Fig. 7:**
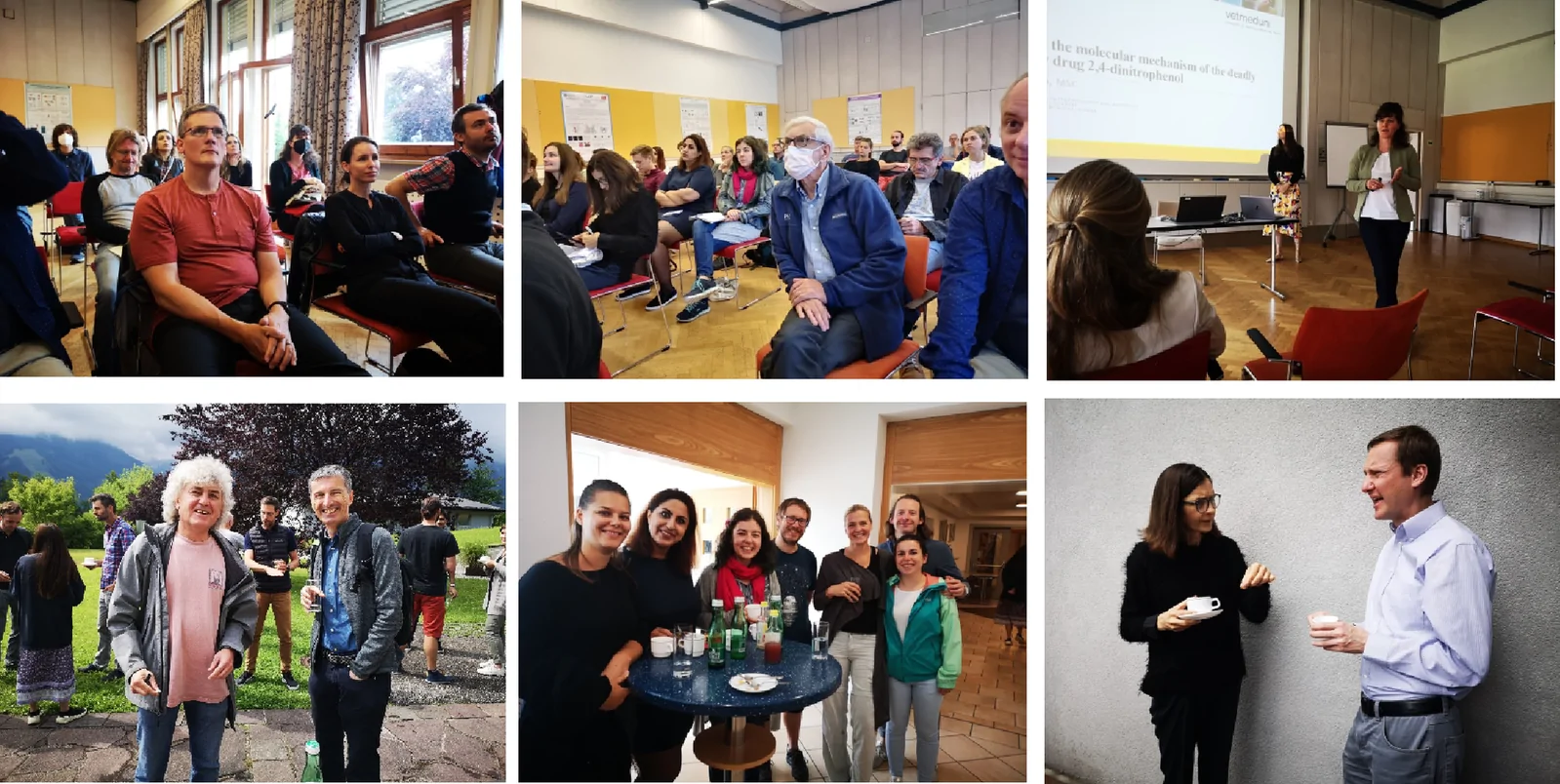
Austrian Biophysical Society Meeting in Strobl, Austria, 9-11th September, 2022. ©Elena E. Pohl, private photo collection..

Motivated by the overwhelming success of the EBSA 2021 meeting the executive committee of Biophysics Austria decided to establish the first meeting of the society separately from ÖGMBT. The first meeting was organized by Peter Pohl (University of Linz) and Birgit Plochberger (Applied University, Linz) and took place in Juni 2022 in Strobl at the Wolfgangsee (Fig. 7). The goal was to bring together Austrian biophysical research groups and to promote more profound cooperation between them. Continuing the newly established tradition of bringing together scientists from different fields of biophysics and from across Austria, the second (2024) and third (2026) Biophysics Austria meetings were held at St. Virgil in Salzburg. They were organized by Bernhard Flucher (Medical University of Innsbruck) and Sandro Keller (Medical University of Graz). Both meetings once again showcased the high quality of research conducted by Austrian biophysics groups, highlighted the strong potential for new collaborations, and reflected the community’s commitment to the society.

## Conclusions and outlook

This year the Austrian Biophysical Society celebrates its 65th anniversary. Recent initiatives of the Executive Committee have focused on actions to increase global visibility of the society. These include the launch of a new society website (https://www.biophysics-austria.com/), designed and maintained by Tony Schmid (Division of Biophysics, Medical University of Graz), and the regular distribution of newsletters to keep members informed about national and international scientific events, funding opportunities, and activities within the Austrian biophysics’ community.

Supporting early-career researchers remains one of society’s highest priorities. Biophysics Austria continues to award travel grants that enable young scientists to participate in major national and international conferences. In addition, Anne Stoib (Johannes Kepler University Linz) has become Austria’s representative in the Young European Biophysical Societies’ Association (yEBSA), a recently established initiative dedicated to strengthening networking, collaboration, and career development among young European biophysicists (https://ebsa.org/young-ebsa/).

Looking ahead, one of our main goals is to further broaden the society by attracting additional research groups working in biophysics and at its interfaces with biology, chemistry, medicine, engineering, and related disciplines.

Above all, Biophysics Austria remains committed to fostering scientific excellence. This is exemplified by the special issue of the *European Biophysics Journal*, *Cutting-Edge Biophysical Research from Austria: Austrian Biophysical Research Groups Present Themselves*, which provides an inspiring overview of the breadth and quality of contemporary biophysical research in Austria (Heiss and Flucher 2025; Kaltenegger et al. 2025; Schneider et al. 2025; Siligan et al. 2025; Costa et al. 2026; Pohl and Jovanovic 2026; Siegl et al. 2026; Sudjarwo and Toca-Herrera 2026; Zhu et al. 2026).

Now, Biophysics Austria enters its seventh decade as an active and constantly growing community, dedicated to support our young researchers and strengthen an expanding international scientific network. Hence, Biophysics Austria is well positioned for an exciting future. Indeed, the best is yet to come.

## Data Availability

No datasets were generated or analysed during the current study.

## Abbreviations

EBJ: European biophysical journal
EBSA: European biophysical societies association
IUPAB: International union for pure and applied biophysics
ÖGB: Österreichische Gesellschaft für Biophysik (Austrian biophysical society)
ÖGMBT: Österreichische Gesellschaft für Molekulare Biowissenschaften und Biotechnologie (Austrian society for molecular biosciences and biotechnology)

## References

[CR1] Broda E, Locker A, Springer-Lederer H (1971) Proceedings of the First European Biophysics Congress, Baden Near Vienna 14–17 Sept. 1971, Austria: Proteins, nucleotides; structure, function, biosynthesis, Verlag d. Wiener Medizinischen Akad

[CR2] Brunner R (1963) [Recent Developments in the Field of Antibiotics (Iii)]. Med Welt 31:1547–1551 14049024

[CR3] Costa P, Maznichenko A, Agmon N, Pohl P (2026) Proton wave propagation along collagen fibers. Eur Biophys J 10.1007/s00249-026-01856-1 PMC13661639 42525057

[CR4] Heiss MC, Flucher BE (2025) Voltage-sensing domains: structural and functional diversity. Eur Biophys J 10.1007/s00249-025-01797-1 PMC13661669 40946106

[CR5] Hinterdorfer P, Baumgartner W, Gruber HJ, Schilcher K, Schindler H (1996) Detection and localization of individual antibody-antigen recognition events by atomic force microscopy. Proc Natl Acad Sci U S A 93(8):3477–3481 10.1073/pnas.93.8.3477 PMC39634 8622961

[CR6] Kaltenegger M, Semeraro EF, Pabst G (2025) Inside the membrane: a closer look using elastic scattering techniques and friends. Eur Biophys J 10.1007/s00249-025-01761-z PMC13661696 40569364

[CR7] Laggner P (1994) X-ray diffraction on biomembranes with emphasis on lipid moiety. Subcell Biochem 23:451–491 10.1007/978-1-4615-1863-1_11 7855880

[CR8] Pohl EE, Jovanovic O (2026) Molecular mechanisms of mitochondrial uncoupling: focus on 2,4-dinitrophenol. Eur Biophys J 10.1007/s00249-026-01827-6 PMC13661760 41838137

[CR9] Schmidt T, Schutz GJ, Baumgartner W, Gruber HJ, Schindler H (1996) Imaging of single molecule diffusion. Proc Natl Acad Sci U S A 93(7):2926–2929 10.1073/pnas.93.7.2926 PMC39736 8610144

[CR10] Schneider MM, Knowles TPJ, Keller S, Krainer G (2025) Microfluidics for protein interaction studies: current methods, challenges, and future perspectives. Eur Biophys J 10.1007/s00249-025-01763-x PMC13661635 40493051

[CR11] Seddon JM, Watts A (2025) EBSA at 40 - an updated history. Eur Biophys J 54(1–2):1–20 10.1007/s00249-024-01730-y 39797926

[CR12] Siegl S, Furthauer S, Schutz GJ (2026) Clustering of the T-cell receptor complex may facilitate T-cell activation by stretching of CD3zeta cytoplasmic tails. Eur Biophys J 10.1007/s00249-026-01861-4 PMC13661662 42507016

[CR13] Siligan C, Gratzl S, Pluhackova K, Goessweiner-Mohr N, Pohl P, Horner A (2025) Cysteine-mediated structural stabilization of the tetrameric GlpF. Eur Biophys J 10.1007/s00249-025-01803-6 PMC13661670 41182398

[CR14] Sudjarwo WAA, Toca-Herrera JL (2026) Multiscale characterization of lipid bilayer disruption induced by Methyl-beta-cyclodextrin (MbetaCD). Eur Biophys J 10.1007/s00249-026-01821-y PMC13661649 41609816

[CR15] von Bertalanffy L (1965) Die Biophysik offener Systeme Naturwissenschaftliche Rundschau 18(12):467–469

[CR16] Zhu R, Oh YJ, Hinterdorfer P (2026) Single molecule force spectroscopy for evaluating inhibitors of SARS-CoV-2 variants of concern. Eur Biophys J 10.1007/s00249-026-01818-7 PMC13661725 41553427

